# Intestinal endometriosis treated by laparoscopic surgery: case series of 5 patients

**DOI:** 10.1186/s40792-020-00811-2

**Published:** 2020-03-10

**Authors:** Hiroka Kondo, Yasumitsu Hirano, Toshimasa Ishii, Kiyoka Hara, Nao Obara, Liming Wang, Masahiro Asari, Takuya Kato, Shigeki Yamaguchi

**Affiliations:** Department of Gastroenterological Surgery, Saitama Medical University International Medical Center, 1397-1 Yamane, Hidaka-shi, Saitama, 350-1298 Japan

**Keywords:** Intestinal endometriosis, Laparoscopic surgery

## Abstract

**Background:**

Intestinal endometriosis is rare and most frequently involves the rectum and sigmoid colon.

**Case presentation:**

Here, we report a case series of 5 patients who underwent laparoscopic resection for intestinal endometriosis. None of the patients developed postoperative complications, and all were discharged at 5–8 days after surgery. The diagnosis of intestinal endometriosis is difficult to obtain before surgery. Only 2 of 5 patients were diagnosed preoperatively. Among 1 of the 2 patients, the symptoms at the time of menstruation were obvious. In patients with submucosal tumors, the preoperative diagnosis can be difficult. Additional imaging examinations at the time of menstruation might be useful for obtaining a diagnosis. D2 dissections were performed for 3 patients, because malignancy could not be ruled out as a preoperative diagnosis. The surgical findings of 1 patient did not appear to be endometriosis. Surgery for intestinal endometriosis usually encounters advanced pelvic adhesions and fibrosis. For patients undergoing sigmoidectomy, the mean operative time was 152 min and mean blood loss was 10 mL. For patients undergoing rectal resection, the mean operative time was 282 min and mean blood loss was 17 mL. Two cases had severe pelvic adhesions, and the residual rectum could not be straightened. Therefore, side-to-side anastomosis was performed. For intestinal endometriosis surgery, flexible planning for the anastomosis method used for residual intestine should be undertaken.

**Conclusion:**

Laparoscopic surgery for intestinal endometriosis was safe but technically difficult, because of fibrosis and adhesions. An accurate diagnosis should be attempted based on the clinical symptoms, imaging findings, and intraoperative findings. The method used for anastomosis should be decided on a case-by-case basis.

## Background

Intestinal endometriosis is rare. It is seen in about 10 to 30% of patients with endometriosis [1] and most frequently involves the rectum and sigmoid colon [2]. Here, we report a case series of 5 patients who underwent laparoscopic resection for intestinal endometriosis.

## Case report

### Case 1: a 49-year-old woman

**Fig. 1:**
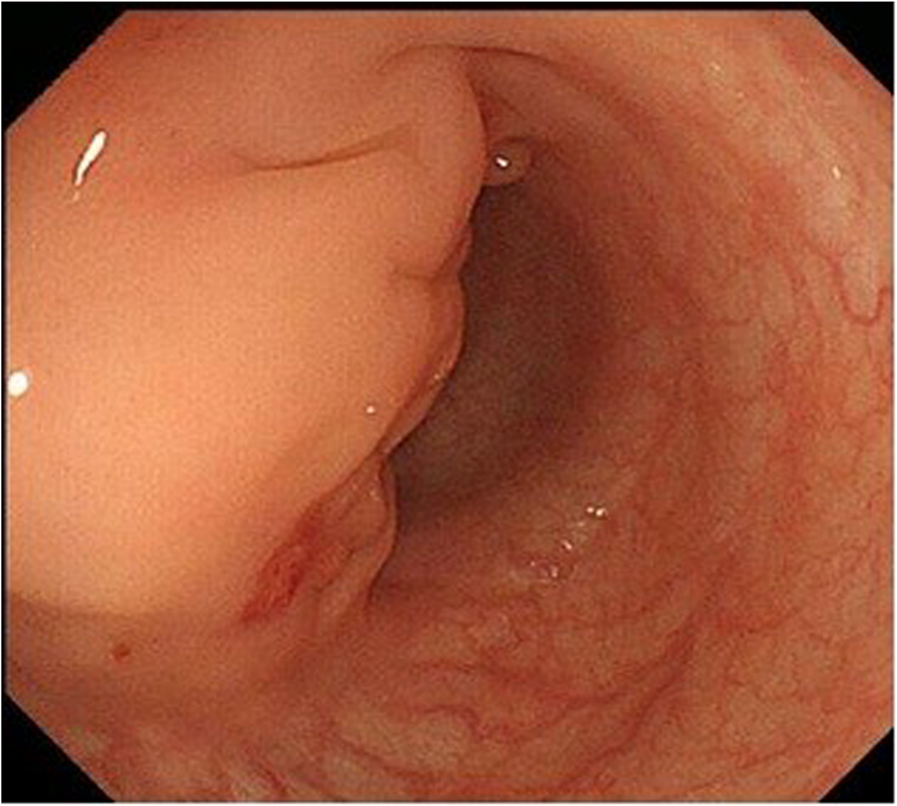
Colonoscopy revealed an extramural mass associated with the sigmoid colon

**Fig. 2:**
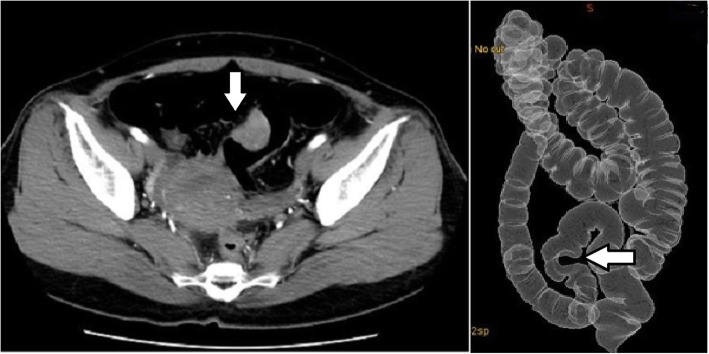
Computed tomography (CT) reveals the thickened wall of the sigmoid colon (arrow), and CT colonography shows the deformed wall in the sigmoid colon (arrow)

After a positive fecal occult blood test (FOBT), colonoscopy revealed an extramural mass in the sigmoid colon (Fig. 1), and the patient was referred to our hospital. Computed tomography (CT) revealed thickening of the wall of the sigmoid colon (Fig. 2). Our preoperative diagnosis was a malignant tumor such as gastrointestinal stromal tumor (GIST). We performed laparoscopic sigmoidectomy. The pouch of Douglas contained severe fibrosis. Side-to-side anastomosis was performed because straightening the rectum was difficult.

### Case 2: a 48-year-old woman

**Fig. 3:**
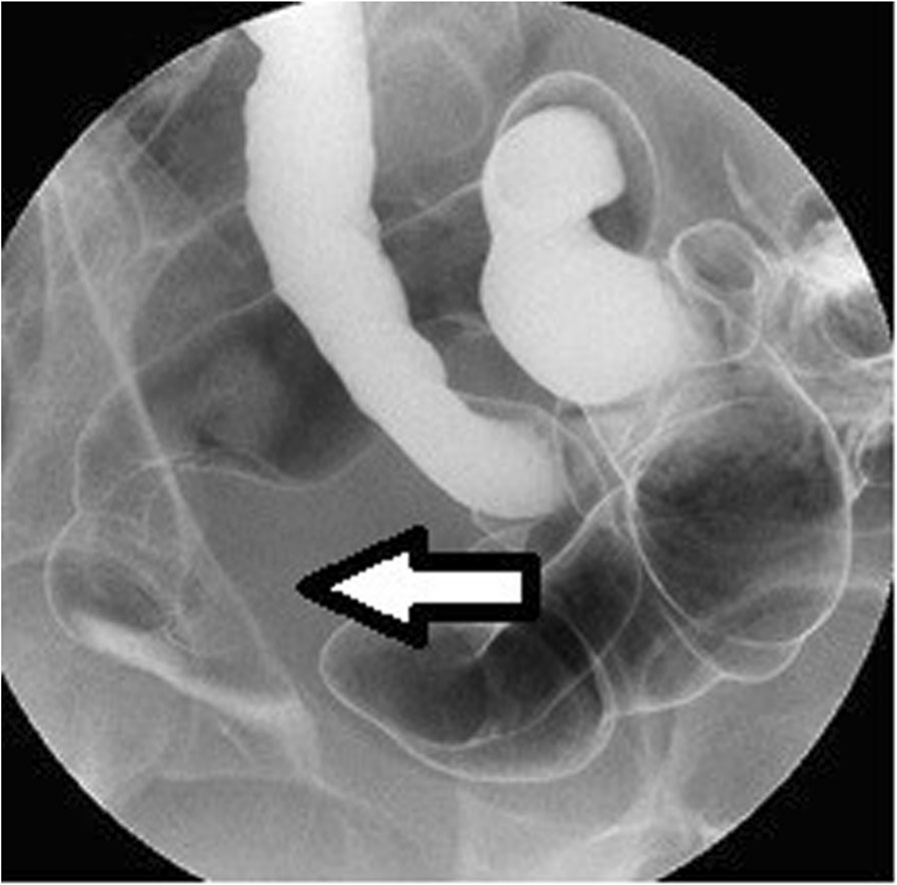
A barium enema examination shows a 40-mm extramural mass in the rectum

**Fig. 4:**
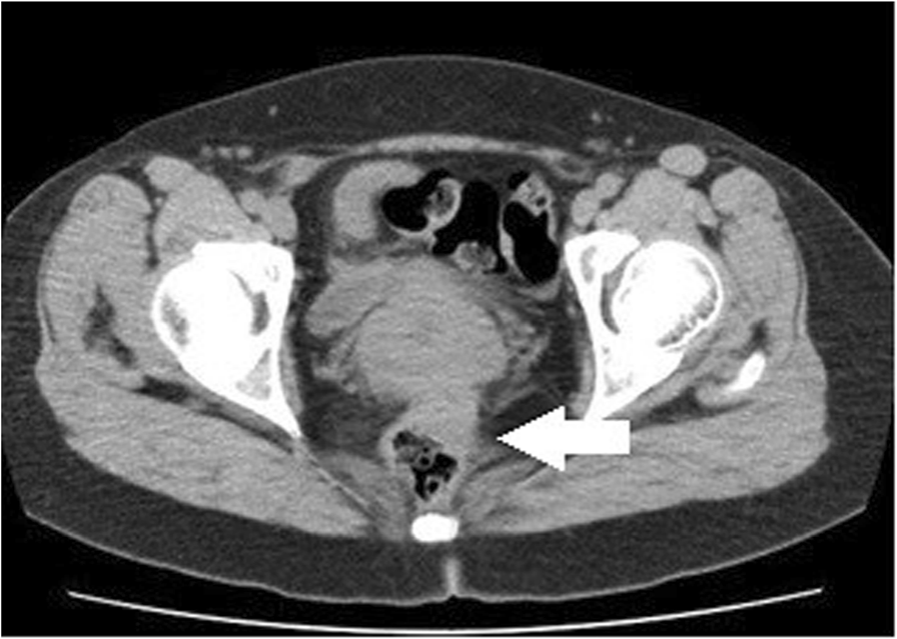
Computed tomography shows the 40-mm mass on the anterior wall of the rectum

**Fig. 5:**
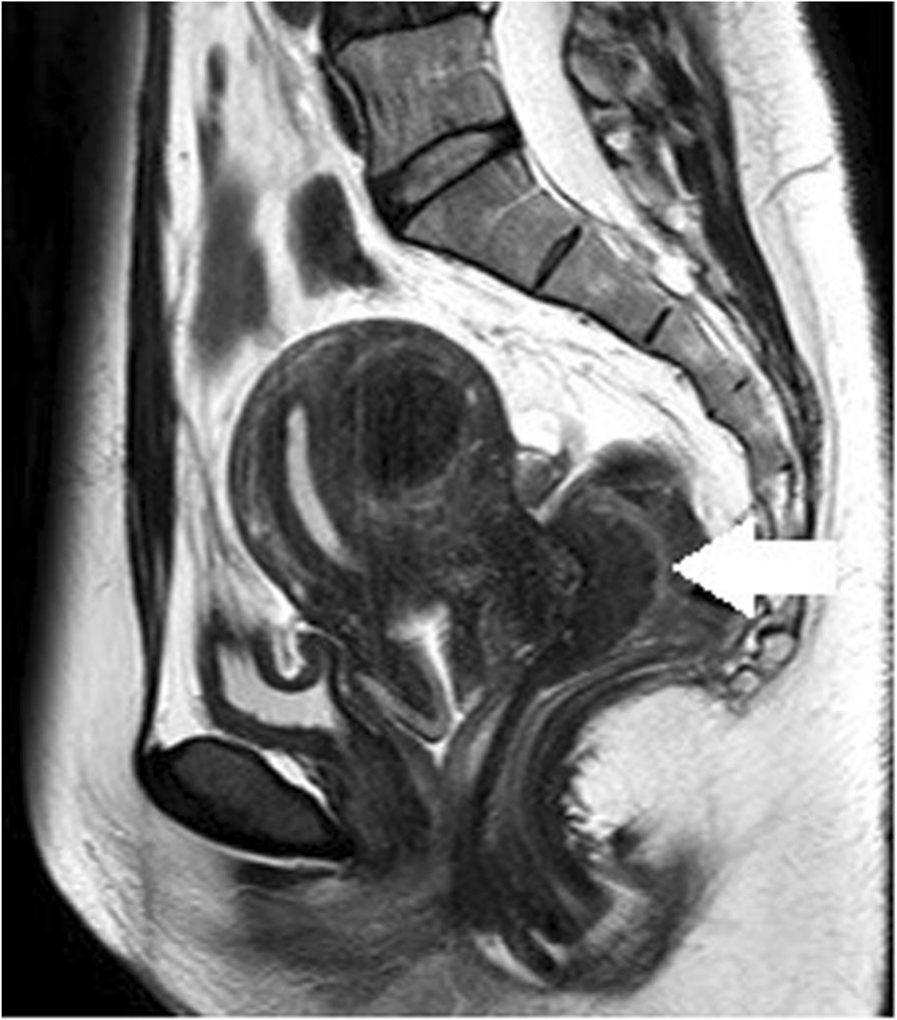
Magnetic resonance imaging (MRI) shows a 40-mm mass on the anterior wall of the rectum

After a positive FOBT, and with a history of constipation, the patient underwent rectal examination, which revealed a palpable mass. A barium enema examination revealed a 40-mm extramural mass in the rectum (Fig. 3), and the patient was referred to our hospital. A colonoscopy was negative for mucosal findings. Computed tomography (CT) and magnetic resonance imaging (MRI) revealed a 40-mm mass on the anterior wall of the rectum (Figs. 4 and 5, respectively). Our preoperative diagnosis was a malignant tumor such as GIST. We performed a low anterior laparoscopic resection and found severe fibrosis in the pouch of Douglas.

### Case 3: a 50-year-old woman

**Fig. 6:**
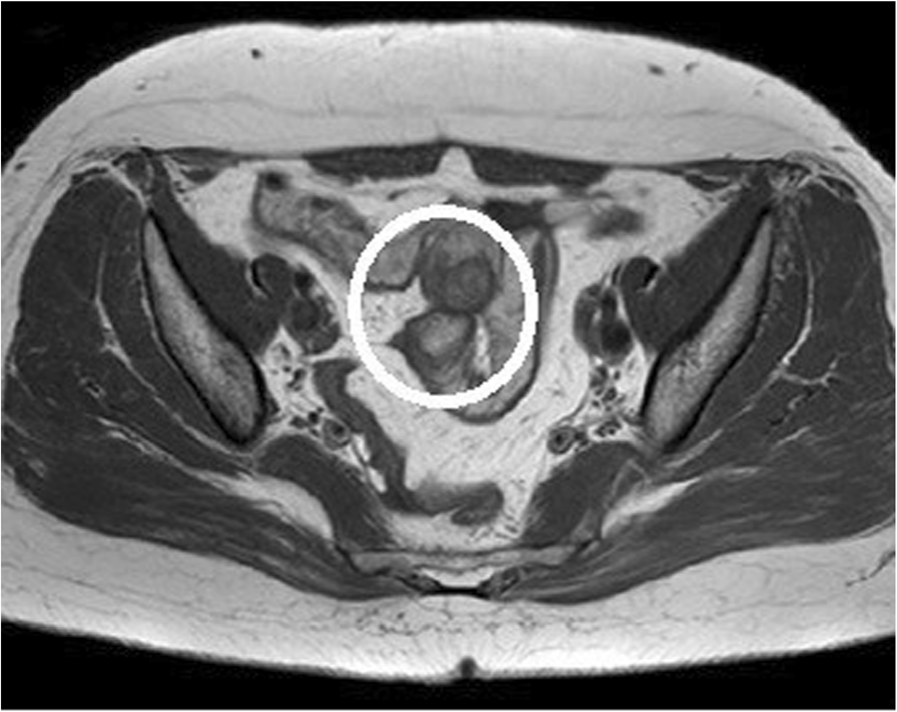
MRI shows a mass at the end of the ileum (circle)

The patient presented at another hospital with a history of 2 to 3 months of abdominal pain. Abdominal ultrasonography revealed a mass at the end of the ileum, and she was referred to our hospital. A colonoscopy revealed a submucosal tumor at the end of the ileum, and we marked the location by a tattoo. CT was unremarkable, but MRI revealed a mass at the end of the ileum (Fig. 6). Our preoperative diagnosis was intestinal endometriosis because symptoms always appear at menstruation. A 30-mm mass was found at the end of the ileum, and a partial small bowel resection and end-to-end anastomosis were performed.

### Case 4: a 37-year-old woman

**Fig. 7:**
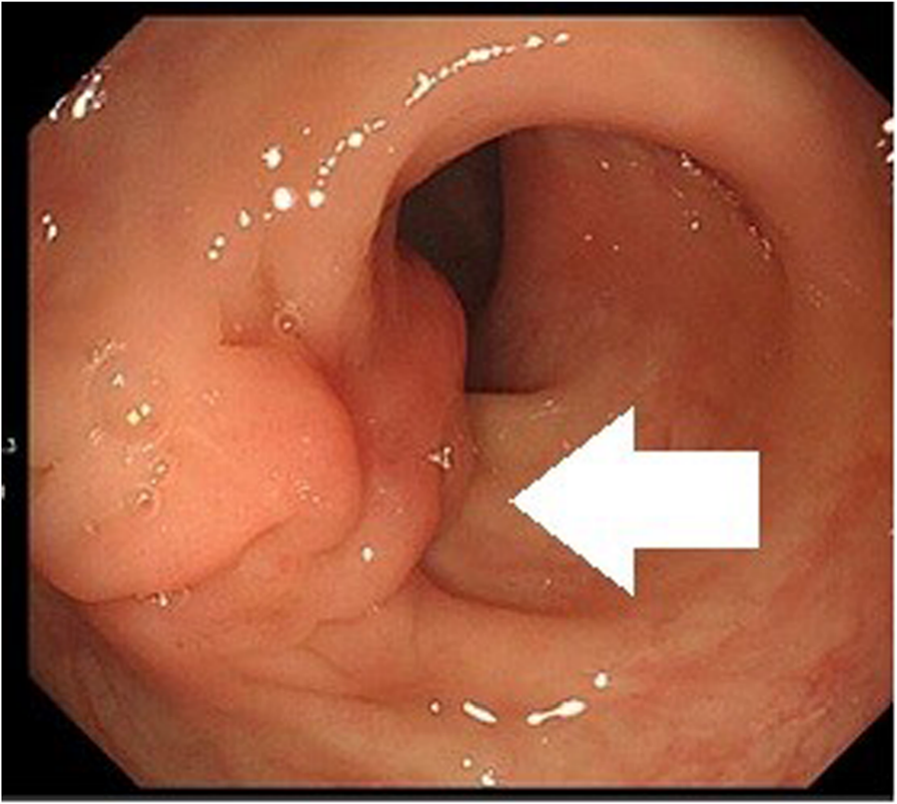
Colonoscopy shows a 25-mm submucosal tumor in the sigmoid colon (arrow)

**Fig. 8:**
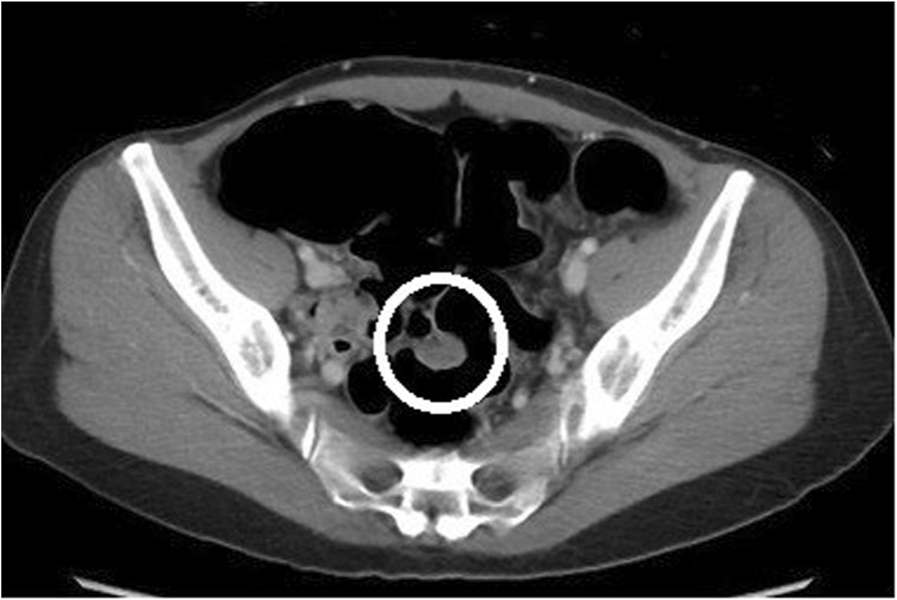
Computed tomography shows a 20-mm mass in the sigmoid colon (circle)

After a positive FOBT, colonoscopy revealed a 25-mm submucosal tumor in the sigmoid colon (Fig. 7), and the patient was referred to our hospital. CT reveal a 20-mm mass in the sigmoid colon (Fig. 8). Our preoperative diagnosis was a malignant tumor such as GIST. We performed a high anterior laparoscopic resection and found severe fibrosis in the pouch of Douglas. We suspected endometriosis.

### Case 5: a 43-year-old woman

**Fig. 9:**
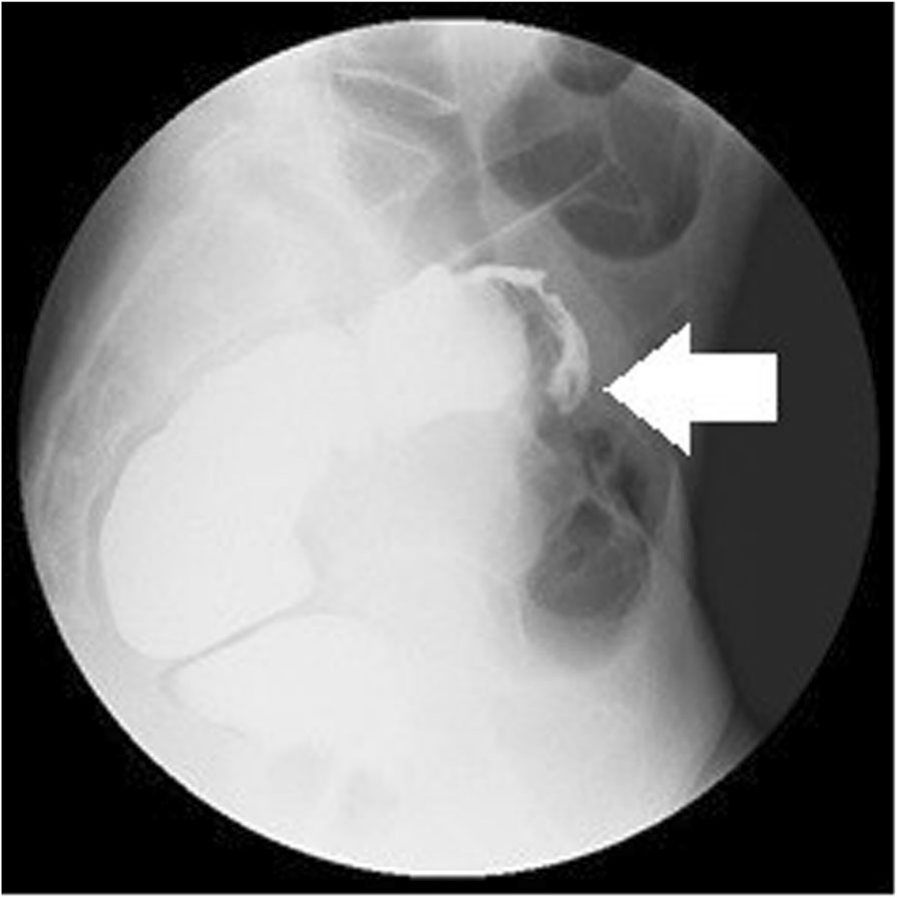
A barium enema examination shows an extramural mass in the sigmoid colon (arrow)

**Fig. 10:**
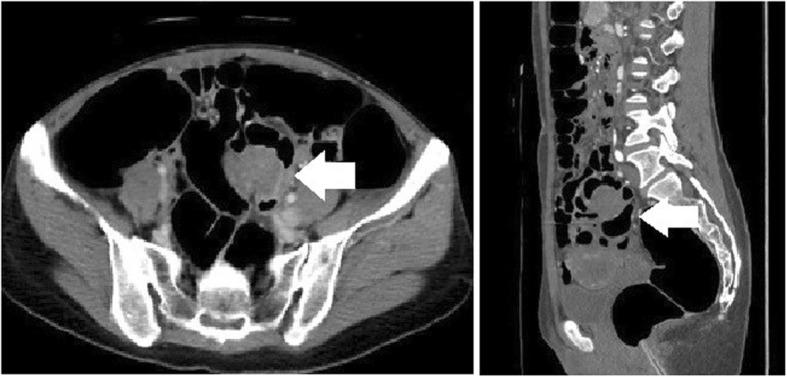
Computed tomography shows the mass in the sigmoid colon (arrow)

The patient presented at another hospital with a 5-year history of constipation. A barium enema examination revealed an extramural mass in the sigmoid colon (Fig. 9), and she was referred to our hospital. A colonoscopy revealed an extramural mass in the sigmoid colon, and the scope could not be passed due to stenosis. CT revealed the mass in the sigmoid colon (Fig. 10). The referral gynecologist had diagnosed endometriosis, and our preoperative diagnosis was intestinal endometriosis. We performed a laparoscopic sigmoidectomy and found severe fibrosis in the pouch of Douglas. Because straightening the rectum was difficult, a side-to-side anastomosis was performed.

Preoperative biopsies were performed in two of the five patients, but none were diagnosed because they were biopsies from the mucosal surface. Although endoscopic ultrasound-guided fine needle aspiration (EUS-FNA) was considered in all cases, all cases were symptomatic and early surgery was requested, so EUS-FNA was not performed.

None of the patients developed postoperative complications, and all patients were discharged at 5–8 days after surgery.

## Discussion

Endometriosis is a general term for endometrial tissue found outside the uterus and is present in 5 to 15% of women of reproductive age [3]. It has been reported that 12 to 37% of patients with endometriosis have intestinal endometriosis [1]. Ectopic endometrial tissue invades and grows in the intestinal tract, causing bleeding, stenosis, and adhesions. The most common site of this disease is said to be the sigmoid colon or rectum [2].

Before surgery, intestinal endometriosis was diagnosed in 2 of the 5 patients. Otherwise, it was difficult to diagnose preoperatively. Among the 2 patients, the clarifying symptom of pain was present at the time of menstruation in only 1 patient.

In case 3, non-menstrual CT showed no findings at the terminal ileum, but MRI performed during menstruation showed a mass. Because of the different modalities, it is not possible to conclude based on this report alone, but it was also important to examine the difference between menstrual and non-menstrual images in diagnostic imaging of intestinal endometriosis. Although no reports on menstrual and non-menstrual imaging have been found in previous reports, the accumulation of future cases is considered important. Obtaining a preoperative diagnosis appears to be difficult for patients with a submucosal lesion; however, Oliveira et al. reported that intestinal endometriosis should be considered in the differential diagnosis of patients with submucosal tumors of the colon [4]. If intestinal endometriosis is established as part of the differential diagnosis, a preoperative diagnosis should not be difficult.

D2 dissection was performed for case nos. 1, 2, and 4; because malignancy could not be ruled out preoperatively. Tumors arising from intestinal endometriosis are rare and are collectively referred to as endometriosis-associated intestinal tumor (EAIT) [5]. Malignant transformation can develop in some cases [6], and metastatic lymph nodes have been observed [7]. Previous reports indicate that 78.7% of endometrial malignancies occur in the ovaries and 21.3% outside the ovaries. Of the latter, the pelvis accounted for 5.7%, the rectovaginal septum 4.3%, the colorectum 4.3%, and the small intestine 0.5% [8].

There have been reports that endometrial tissue has also been found in regional lymph nodes at the time of the resection of the intestine involved with endometriosis; however, the relationship between lymph nodes containing endometrial lesions and the malignant transformation of endometrial lesions in the intestine has not been clarified, and carcinomas associated with intestinal endometriosis are rare [9, 10]. Some believe that lymphadenectomy is not so effective for endometriosis [11]. If there is no suspicion of a malignant tumor, lymph node dissection is unnecessary, even if the lymph nodes are involved. If malignancy cannot be ruled out, excision of the intestinal tract with lymph node dissection should be considered.

The surgical findings of 1 of our 5 cases did not suggest endometriosis. The surgical findings of surgery for intestinal endometriosis often include advanced pelvic adhesions and fibrosis. These findings are thought to be caused by endometrial tissue, which undergoes fibrosis, and metaplasia of smooth muscle extending throughout the muscular layer to the subserosal layer of the intestinal tract and repeatedly causing bleeding and inflammation along with the menstrual cycle [12].

Rocha et al. have evaluated laparoscopic surgery in endometriosis surgery with colectomy and report that it is also effective in alleviating abdominal symptoms and preserving fertility [13]. For patients undergoing sigmoidectomy, the mean operative time was 152 min and mean blood loss was 10 mL. For patients undergoing rectal resection, the mean operative time was 282 min and mean blood loss was 17 mL. Two cases had severe pelvic adhesions, and the residual rectum could not be straightened. Therefore, side-to-side anastomosis was performed. For intestinal endometriosis surgery, flexible planning for the anastomosis method for a residual intestine should be undertaken.

## Conclusion

Laparoscopic surgery for intestinal endometriosis was safe but technically difficult, because of fibrosis and adhesions. An accurate diagnosis should be attempted based on the clinical symptoms, imaging findings, and intraoperative findings. The method used for anastomosis should be decided on a case-by-case basis.

## Data Availability

Data sharing is not applicable to this article, since datasets were neither generated nor analyzed for the case series.

## Abbreviations

CT: Computed tomography
EUS-FNA: Endoscopic ultrasound-guided fine-needle aspiration
FOBT: Fecal occult blood test
GIST: Gastrointestinal stromal tumor
MRI: Magnetic resonance imaging
